# Diagnostic accuracy of the interferon-gamma release assay in acquired immunodeficiency syndrome patients with suspected tuberculosis infection: a meta-analysis

**DOI:** 10.1007/s15010-022-01789-9

**Published:** 2022-03-06

**Authors:** Hao Chen, Atsushi Nakagawa, Mikio Takamori, Seitarou Abe, Daisuke Ueno, Nobuyuki Horita, Seiya Kato, Nobuhiko Seki

**Affiliations:** 1 Department of Internal Medicine, Teikyo University Graduate School of Medicine, Tokyo, Japan; 2 Department of Respiratory, Kobe City Medical Center General Hospital, Kobe, Japan; 3 Department of Respiratory, Tokyo Metropolitan Tama Medical Center, Tokyo, Japan; 4 Department of Respiratory, Niigata Prefectural Shibata Hospital, Niigata, Japan; 5 Department of Emergency Medicine, Kawasaki Medical School Hospital, Okayama, Japan; 6 Department of Pulmonology, Yokohama City University, Yokohama, Japan; 7 Research Institute of Tuberculosis, Japan Anti-Tuberculosis Association, Tokyo, Japan; 8 Department of Oncology, Teikyo University Hospital, 2-11-1 Kaga, Itahashi, Tokyo 173-8606 Japan

**Keywords:** Interferon-gamma release assay, Tuberculosis, People living with HIV, Sensitivity, Specificity

## Abstract

**Purpose:**

The diagnostic accuracy of the interferon-gamma release assay (IGRA) in immunosuppressed patients remains unclear.

**Methods:**

A systematic review and meta-analysis were performed for diagnostic test accuracy of IGRA in tuberculosis (TB) infection among people living with HIV (PLWHIV). Summary estimates of sensitivity and specificity were calculated using both univariate and bivariate models.

**Results:**

The meta-analysis included 45 of the 1,242 first-screened articles. The total number of PLWHIV was 6,525; 3,467 had TB disease, including 806 cases of LTBI and 2,661 cases of active TB. The overall diagnostic odds ratio (DOR) of IGRA in the diagnosis of TB disease was 10.0 (95% confidence interval (CI) 5.59, 25.07), with an area under the curve (AUC) of 0.729. The DOR was better for QFT (14.2 (95%CI 4.359, 46.463)) than T-SPOT (10.0 (95%CI 3.866 26.033)). The sensitivity and specificity of QFT and T-SPOT were 0.663 (95%CI 0.471, 0.813), 0.867 (95%CI 0.683 0.942), and 0.604 (95%CI 0.481, 0.715), 0.862 (95%CI 0.654, 0.954), respectively, in the bivariate model. The sensitivity of IGRA in the diagnosis of LTBI was 0.64 (95%CI 0.61, 0.66).

**Conclusion:**

IGRA was useful in the diagnostic of TB disease in PLWHIV, and QFT showed a better tendency of DOR than T-SPOT. IGRA showed a limited effect to rule out LTBI in PLWHIV.

**Supplementary Information:**

The online version contains supplementary material available at 10.1007/s15010-022-01789-9.

## Introduction

Individuals infected with *Mycobacterium tuberculosis* (*Mtb*) may develop symptoms and signs of active tuberculosis (ATB) or may stay in latent tuberculosis infection (LTBI) which have no clinical evidence of the active disease [1]. Mtb is the leading cause of opportunistic infection involved in the death of people living with human immunodeficiency virus (PLWHIV) [2], while the diagnosis is further problematic due to its paucibacillary nature. In addition, human immunodeficiency virus (HIV) infection may cause respiratory problems that can mimic tuberculosis clinically and/or radiologically. An early diagnosis in this group is thus important.

Until recently, the tuberculin skin test (TST) has been the only method to test for latent infection with Mtb*.* The TST has well-known strengths and limitations by measuring the delayed type hypersensitivity response to intradermal injection of purified protein derivative [3]. Whereas the TST encompasses antigens recognized by a vast pool of circulating T lymphocytes, the two interferon-gamma (IFN-γ) release assays (IGRAs), the QuantiFERON-TB® assay (Cellestis Limited, Victoria, Australia) and T-SPOT-TB® (Oxford Immunotec, London, UK), focus on interferon-gamma responses to epitopes from two specific antigens which is associated with Mtbs complex, namely early secretory antigenic-6 (ESAT-6) and culture filtrate protein-10 (CFP-10). No direct tests for LTBI, and therefore no gold standards, are available with which to compare LTBI test characteristics [4]. IGRA rather than TST was recommended by the Centers for Disease Control and Prevention (CDC) in individuals 5 years or older upon the likelihood of infection with Mtb and the likelihood of progression to TB disease if infected [1].

For the IGRA or TST to reliably rule out a diagnosis of Mtb infection and thus TB disease, the sensitivity of the test must be very high (>95%) [5]. The sensitivity and specificity of IGRAs compared with the TST in active TB have been examined in several studies, varying in value and quality [6]. IGRA have a better predictive ability than tuberculin skin tests. Individuals who are positive on an IGRA might benefit from preventive treatment, but those who are positive by TST probably will not [7]. The pooled sensitivity (95% confidence interval (CI)) of QFT and T-SPOT, T-SPOT, and TST was: 80% (75–84%), 81% (78–84%), and 65% (61–68%), respectively, in the previous meta-analysis and the sensitivity of IGRAs was too low to support their use as rule-out tests for active TB [8]. PLWHIV represents a group at higher risk of reactivating LTBI. Furthermore, immunosuppression can lower the sputum bacillary load, making the diagnosis of ATB by microscopy more challenging [9]. In PLWHIV, the performance of IGRAs is not as reliable as previously measured in the general population [10]. The diagnostic accuracy of IGRAs in PLWHIV was necessary to answer the question of timely TB diagnosis and the medical community is cautious in interpretation of IGRAs’ results. This study aimed to evaluate the contribution of IGRAs to the diagnosis of TB disease in PLWHIV.

## Methods

### Study overview

This systematic review and meta-analysis of diagnostic test accuracy was prepared following standard guidelines for systematic reviews of diagnostic test accuracy and registered on the website of the University Hospital Medical Information Network Clinical Trials Registration (UMIN000045715) [11, 12]. Due to the nature of this study, approval of the Institutional Review Board was waived.

### Study search

Four major online databases, PubMed, Web of Science, Cochrane, and Embase, were searched (September 30, 2021). The following search strategy was used for PubMed: (((interferon-γ release assay) OR (interferon-gamma release assay)) OR (IGRA)) AND ((((HIV) OR (Human Immunodeficiency Virus)) OR (acquired immunodeficiency syndrome)) OR (AIDS)).

Two authors (HC and AN) independently screened the titles and abstracts and carefully evaluated the full text to select eligible articles; in cases of discrepancy, they reached a consensus through discussion. Review articles and included original articles were hand-searched (HC and AN) for additional research papers that met the inclusion criteria.

### Study selection

Full articles, brief reports, and conference abstracts published in any language that provided data for sensitivity and specificity of IGRA to diagnose TB were included. An article that provided data of both sensitivity and specificity was included in the bivariate analysis [11]. An article that provided data of either sensitivity or specificity was included in the univariate analysis. A case–control study design that consisted of patients with or without TB disease was accepted, though a case–control design may be considered to have a risk of bias according to Quality Assessment of Diagnostic Accuracy Studies-2 (QUADAS-2) [13].

The target population was PLWHIV with TB and LTBI co-infection. The diagnostic criterion for ATB was sputum culture-positive for TB or detection of nucleic acids, both DNA and RNA, which are specific to Mycobacterium tuberculosis, by amplification techniques such as polymerase chain reaction. LTBI is a subclinical mycobacterial infection defined on the basis of cellular immune response to mycobacterial antigens [14]. TST and IGRA are currently used to establish the diagnosis of LTB. The diagnostic criteria for LTBI were either positive on IGRA or TST or a radiograph without clinical findings of active TB. The target IGRA test included T-SPOT and QFT.

### Outcomes

Sensitivity, specificity, area under the curve (AUC), and the diagnostic odds ratio were evaluated in studies with both sensitivity and specificity. Univariate analysis was conducted for studies with either sensitivity or specificity. Only data from the 3rd and 4th generations of QFT (QuantiFERON-TB-Gold In-Tube and QuantiFERON-plus) were included for test accuracy in this meta-analysis. Studies with test accuracy of the T-SPOT were all enrolled in this analysis. Indeterminate IGRA results were classified as false-negative results.

### Data extraction

Two review authors, HC and AN, independently extracted data, including the name of the first author, publication year, publication country, numbers of patients with positive results, numbers of patients evaluated, and QUADAS-2-related information. Risk of bias was appraised by QUADAS-2 in each study [13].

### Statistics

A bivariate model was used to obtain pooled sensitivity and specificity and to draw a summary receiver-operating characteristic curve (SROC) [15]. The diagnostic odds ratio (DOR) was obtained by the DerSimonian–Laird random model. The DOR was calculated by the “madauni” command (“netmeta” package of R project, Gerta Rücker, Denmark). Sensitivity, specificity, and AUC were pooled by the “reitsma” command (“netmeta” package of R project, Gerta Rücker, Denmark). AUCs were interpreted as follows: ≥0.97, excellent; 0.93–0.96, very good; 0.75–0.92, good; and 0.5–0.74, fair [16]. The threshold for significance was set at 0.05. Heterogeneity evaluated using *I*^2^ statistics was interpreted as follows: *I*^2^ = 0%, no heterogeneity; *I*^2^ > 0% but <25%, minimal heterogeneity; *I*^2^ ≥ 25% but <50%, mild heterogeneity; *I*^2^ ≥ 50% but <75%, moderate heterogeneity; and *I*^2^ ≥ 75%, strong heterogeneity [17].

## Results

### Study search and study characteristics

A total of 1,242 articles, including 1,239 articles through database search and 3 articles by hand search, were identified; 937, 192, and 47 articles were left after removing duplication, screening, and full-article reading, respectively (Supplementary Figure S1). Finally, 45 reports, comprising 42 full-length articles and 3 conference abstracts, were included (Table 1) [2, 5, 18–60]. All were written in English. Prospective study designs were adopted in 34 articles, and the other 11 were retrospective studies. Of the 47 reports, six were from South Africa, five were from the USA, four were from China, Italy, and the UK, and two were from Brazil and India. Of the 6,525 PLWHIV were enrolled in this study, 3,467 had TB disease, including 806 cases of LTBI and 2,661 cases of ATB. Nine studies discussed diagnostic accuracy including both T-SPOT and QFT, and 22 and 12 studies discussed the test accuracy of only the QFT or the T-SPOT, respectively. Only five studies discussed test accuracy in children, and two studies checked IGRAs in all populations. The remaining 38 studies checked IGRAs in adults, including two studies that checked IGRAs in women only.

**Table 1:** Background characteristics of enrolled studies

Author/year	Country	Types of TB	Type of article	Nature of study	Adult	IGRA	TB patients	All patients
Aabye 2009	Tanzania	AT	FA	Retro	Adult	QFT-GIT	161	161
Adams 2019	South Africa	LTBI	FA	Retro	Adult	Q&T	496	496
Cai 2014	China	AT	FA	Retro	Adult	T-SPOT	100	100
Cattamanchi 2010	USA	AT	FA	pros	Adult	T-SPOT	112	212
Chee 2008	Singapore	AT	FA	retro	Adult	Q&T	280	280
Chen 2011	China	AT	FA	pros	Adult	T-SPOT	38	147
Clark 2007	UK	AT	FA	pros	Adult	T-SPOT	30	30
Davies 2009	South Africa	AT	FA	pros	Children	T-SPOT	60	109
Dheda 2009	South Africa	AT	FA	pros	Adult	Q&T	20	20
Elzi 2011	Switzerland	LTBI	FA	pros	Adult	T-SPOT	64	64
Fujita 2011	Japan	AT	FA	pros	Adult	QFT-GIT	9	107
Garcia-Gasalla 2013	Spain	AT	FA	pros	Adult	QFT-GIT	118	118
Hormi 2018	France	AT	FA	pros	Children	QFT-GIT	24	24
Idh 2010	Sweden	AT	FA	pros	Adult	QFT-GIT	69	69
Jiang 2009	China	AT&LTBI	FA	pros	Adult	T-SPOT	100	100
Jonnalagadda 2013	USA	AT	FA	retro	Adult(W)	T-SPOT	9	9
Kabeer 2011	India	AT	FA	pros	Adult	QFT-GIT	105	105
Kaswandani 2018	Indonesia	TB	CA	retro	Children	QFT-GIT	10	10
Khawcharoenporn 2015	Thailand	LTBI	FA	pros	Adult	QFT-GIT	36	36
Klautau 2018	Brazil	LTBI	FA	retro	Adult	QFT-GIT	84	84
Kussen 2016	Brazil	LTBI	FA	pros	Adult	QFT-GIT	25	25
LaCourse 2017	USA	AT	FA	pros	Adult (W)	QFT-GIT	100	100
Lavender 2011	UK	AT	CA	retro	Adult	QFT-GIT	66	326
Lee 2019	Korea	TB	FA	pros	Adult	T-SPOT	25	62
Legesse 2010	Ethiopia	AT	FA	pros	Adult	QFT-GIT	50	50
Leidl 2010	Uganda	AT	FA	retro	Adult	Q&T	19	19
ling 2011	Canada	AT	FA	pros	Adult	Q&T	127	127
Lundtoft 2017	Ghana	AT	FA	pros	Children	QFT-GIT	25	25
Markova 2009	Bulgaria	AT	FA	pros	Adult	Q&T	13	90
Oni 2010	UK	AT	FA	pros	Adult	T-SPOT	85	85
Petruccioli 2020	Italy	AT	FA	pros	Adult	QFT-plus	32	32
Pettit 2020	USA	LTBI	FA	pros	All	Q&T	81	1520
Raby 2008	Zambia	AT	FA	pros	Adult	QFT-GIT	96	96
Rangaka 2012	South Africa	LTBI	FA	pros	Adult	QFT-GIT	50	50
Sanogo 2020	Burkina	AT	FA	pros	Children	QFT-GIT	29	58
Sattah 2012	USA	AT	CA	retro	Adult	T-SPOT	9	9
Sauzullo 2010	Italy	AT	FA	pros	Adult	QFT-GIT	30	194
Sauzullo 2014	Italy	AT	FA	pros	Adult	QFT-GIT	44	44
Stavri 2009	Romania	AT	FA	pros	All	QFT-GIT	36	36
Takwoingi 2019	UK	AT	FA	retro	Adult	Q&T	385	911
Tsiouris 2006	South Africa	AT	FA	pros	Adult	QFT-GIT	36	36
Vanini 2012	Italy	AT	FA	pros	Adult	QFT-GIT	58	58
Veldsman 2009	South Africa	AT	FA	pros	Adult	QFT-GIT	30	60
Vincenti 2007	India	AT	FA	pros	Adult	Q&T	45	111
Yu 2013	China	AT	FA	pros	Adult	T-SPOT	46	120

*TB* tuberculosis disease, *AT* active TB; *LTBI* latent tuberculosis infection; *IGRA* interferon-gamma release assay, *QFT-GIT* QuantiFERON-TB-Gold In-Tube, *T-SPOT* T-SPOT.TB, *Q&T* QuantiFERON-TB-Gold In-Tube and T-SPOT.TB

### Diagnostic accuracy of IGRAs in TB

**Fig. 1:**
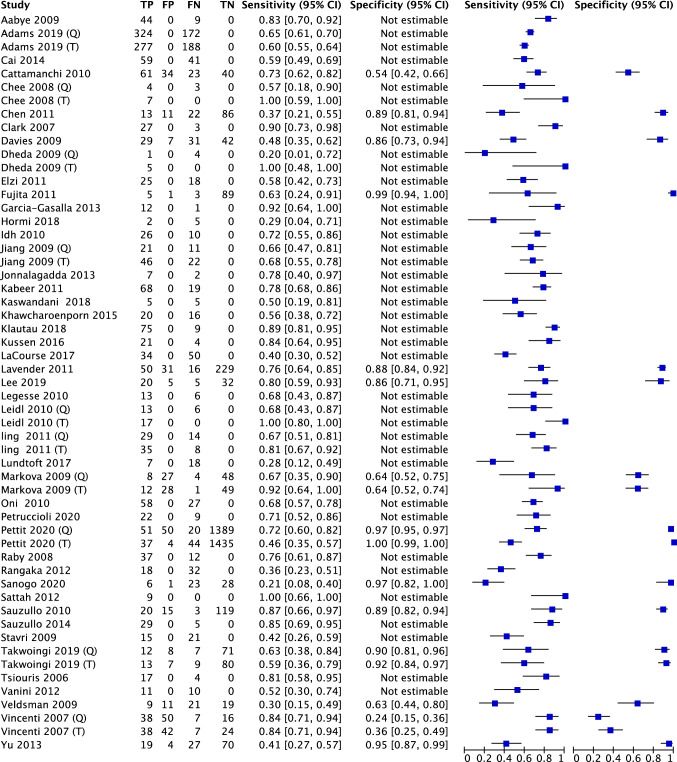
Forest plot of all enrolled studies including IGRA test accuracy. Pooled sensitivity and specificity are 0.65 (95%CI 0.63, 0.66) and 0.92 (95%CI 0.91, 0.93), respectively

Nineteen studies checked the diagnostic accuracy of IGRAs with both sensitivity and specificity. Thirty-nine studies checked the sensitivity of T-SPOT or QFT in PLWHIV with suspected TB. The univariate analysis of IGRAs in PLWHIV showed sensitivity and specificity of 0.65 (95%CI 0.63, 0.66) and 0.92 (95%CI 0.91, 0.93), respectively (Fig. 1).

### Diagnostic accuracy of IGRAs in ATB

**Fig. 2:**
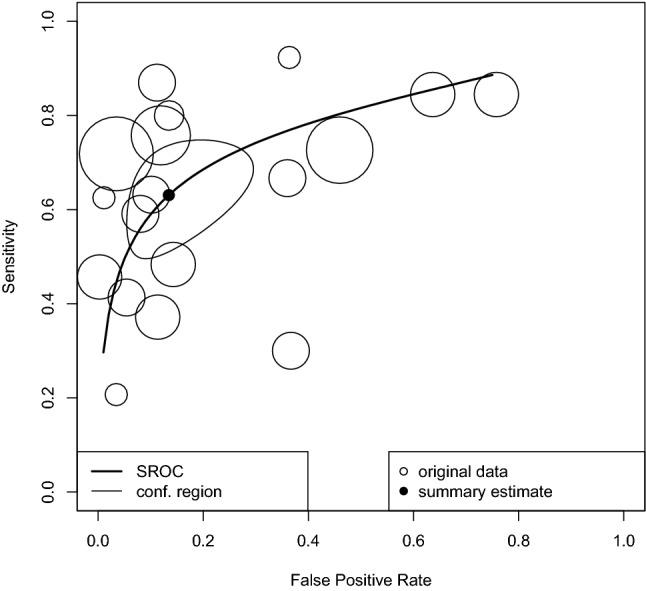
Diagnostic accuracy of IGRAs in AIDS patients

The diagnostic accuracy of IGRAs in ATB was conducted in 49 studies, including in 5,430 participants. On univariate analysis, the sensitivity and specificity were 0.66 (95%CI 0.63, 0.68) and 0.92 (95%CI 0.91, 0.93), respectively (Supplementary Figure S2). On bivariate analysis of the test accuracy of IGRAs in 18 studies, the DOR was 11.84 (95%CI 5.59, 25.07; *I*^2^ = 0%), with AUC of 0.779. This AUC value suggests that IGRA had “good” diagnostic test accuracy for TB disease (Fig. 2) [16]. Using the data from 45 studies of 7,120 specimens, the summary estimates of sensitivity and specificity were 0.631 (95%CI 0.523, 0.727) and 0.866 (95%CI 0.744, 0.934), respectively.

### Diagnostic accuracy of IGRAs in LTBI

Since there was no gold standard to diagnose LTBI, seven studies discussed the sensitivity of IGRA in PLWHIV with different diagnostic standards. Five studies defined LTBI by LTBI risk and at least one positive test (TST or IGRA), without clinical evidence of active TB. Two studies calculated sensitivity from supposed presence of LTBI. The univariate analysis yielded a sensitivity of 0.64 (95%CI 0.61, 0.66) in 1,267 patients (Supplementary Figure S3).

### Diagnostic accuracy of QFT in ATB

**Fig. 3:**
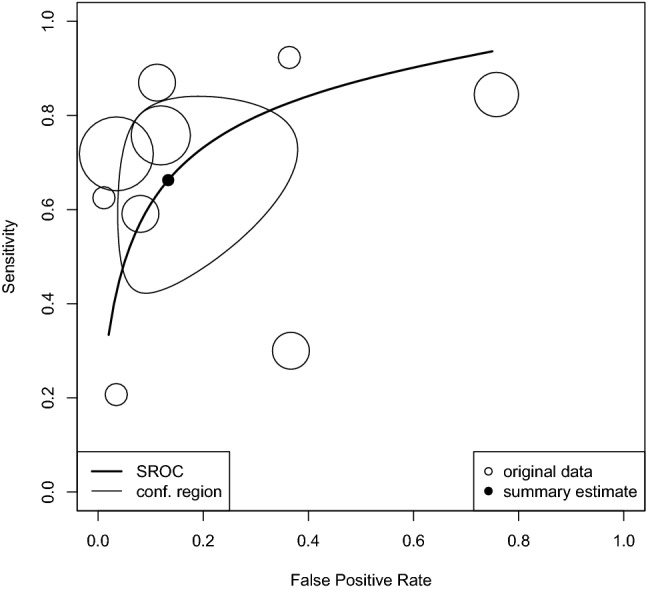
Diagnostic accuracy of QFT in AIDS patients

Data of 2,519 samples from nine reports suggested a DOR of 14.2 (95%CI 4.36, 46.46; *I*^2^ = 0%) and an AUC of 0.822, which means that QFT had “good” diagnostic test accuracy for TB disease (Fig. 3). The summary estimates of sensitivity and specificity were 0.663 (95%CI 0.471, 0.813) and 0.867 (95%CI 0.683 0.942), respectively. The univariate analyses showed sensitivity and specificity of 0.66 (95%CI 0.63, 0.69) and 0.91 (95%CI 0.90, 0.92), respectively, in 27 studies of 3,369 cases of ATB disease in PLWHIV (Supplementary Figure S4).

### Diagnostic accuracy of T-SPOT in ATB

**Fig. 4:**
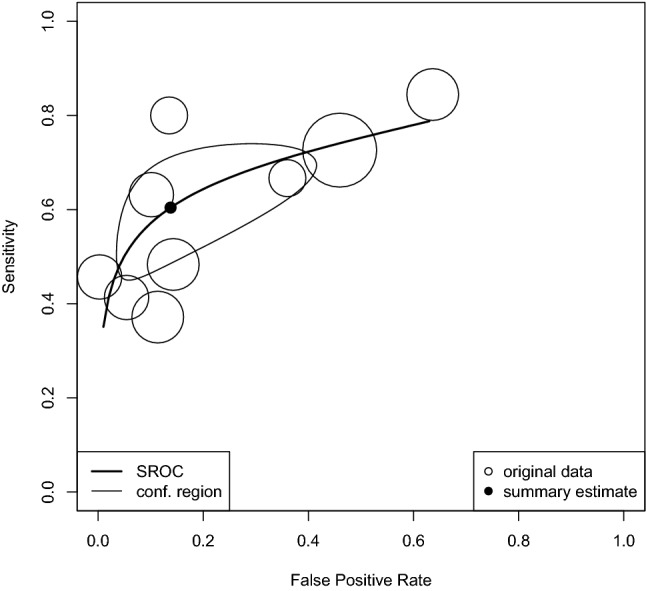
Diagnostic accuracy of T-SPOT in AIDS patients

Nine studies were included in the bivariate analysis of test accuracy with 2,397 samples that yielded a DOR of 10.0 (95%CI 3.87 26.03; *I*^2^ = 2.6%) and an AUC of 0.729. This AUC suggested that T-SPOT had “good” diagnostic test accuracy for TB (Fig. 4) [16]. The summary estimates of sensitivity and specificity were 0.604 (95%CI 0.481, 0.715) and 0.862 (95%CI 0.654, 0.954), respectively. The univariate analysis showed that the sensitivity and specificity of T-SPOT were 0.65 (95%CI 0.62, 0.68) and 0.93 (0.92, 0.94), respectively, in 16 studies of 2,810 patients of TB disease in PLWHIV (Supplementary Figure S5).

### Diagnostic accuracy of QFT and T-SPOT in LTBI

The sensitivity of QFT and T-SPOT in the diagnosis of LTBI was 0.66 (95%CI 0.63, 0.70) and 0.60 (95%CI 0.56, 0.64), respectively (Supplementary Figure S6, S7). The specificity of QFT and T-SPOT was not estimable.

The risk of bias is shown in Supplementary Figure S8. There were 2 studies with unclear patient selection bias, and 7 studies had a high risk of a reference standard issue. No study showed bias in patient selection applicability concerns, index test, index test applicability concerns, reference standard applicability concerns, and flow and timing.

## Discussion

The diagnostic test accuracies of the IGRA QFT and T-SPOT were systematically reviewed. Based on the analysis, QFT showed a better DOR and AUC than T-SPOT in the diagnosis of TB disease, and both of them showed “good” diagnostic accuracy. This systematic review and meta-analysis provided evidence supporting the use of IGRAs in the diagnosis of TB disease in PLWHIV, as in the current guidelines [1, 61]. Although different diagnostic standards were used in seven studies discussing the sensitivity of IGRAs in PLWHIV, IGRAs showed a similar result, with an average sensitivity of 0.64. It was difficult to use IGRAs to rule out a diagnosis of TB disease due to the low sensitivity in PLWHIV. No heterogeneity was observed in the bivariate analysis of IGRA and QFT, and only minimal heterogeneity was confirmed for T-SPOT, which supported the conclusion of this study.

The specificity of QFT and T-SPOT in diagnosis of ATB in PLWHIV was 0.867 (95%CI 0.683 0.942) and 0.862 (95%CI 0.654, 0.954), respectively. The pooled specificity of QFT, T-SPOT, and TST was: 79% (95%CI 75–82%), 59% (95%CI 56–62%), and 75% (95%CI 72–78%), respectively, reported by a previous study [8]. IGRAs and TST have similar (but poor) ability to identify patients with LTBI at risk of developing active TB disease. Both tests may be used in patients where the risk of progression to active TB disease is high and the disease sequelae potentially severe [62]. Compared with the general population, the sensitivity of IGRAs revealed a higher diagnostic accuracy. The improved specificity of IGRAs, however, may reduce the number of patients requiring preventative therapy.

QFT-Plus was more useful than QFT-GIT for the diagnosis of TB infection in all patients, including those who were elderly and/or immunocompromised [63]. Only one study showed data of the diagnostic accuracy in PLWHIV, and the results of 30/31 studies used data of QFT-GIT. However, so far, there was high agreement (>95%) between the QFT-Plus and QFT-GIT [64, 65], QFT-plus might be better than T-SPOT in the diagnosis of TB disease in PLWHIV, but there is limited evidence to support this conclusion. IGRAs and TST are currently used to diagnose candidates for preventive LTBI therapy. The risk of TB disease in patients with an immunocompromised medical condition is greater than that in the general population. Although an increasing number of studies have demonstrated that IGRAs promoted the diagnosis of LTBI because of better specificity, there was still a high false-positive rate in this study of PLWHIV.

There were several limitations in this study. First, almost all of the included studies were two-gate study designs, and a few studies discussed both sensitivity and specificity. The potential for a high risk of selection bias exists. Second, the tuberculosis burden was different in countries, and sub-group analysis was not conducted in different settings. Third, the number of CD4 T cells might affect IGRA test accuracy in PLWHIV. Due to different classifications, only a limited number of studies could perform sub-group analyses.

## Conclusion

IGRA was useful in the diagnosis of TB disease in PLWHIV, and QFT showed a better tendency of DOR than T-SPOT. IGRAs showed a limited effect to rule-out LTBI in PLWHIV.

## Supplementary Information

Below is the link to the electronic supplementary material.

Supplementary file1 (DOCX 1451 KB)

## Data Availability

The raw data are available by email on reasonable request to the corresponding author at nseki@med.teikyo-u.ac.jp.
