# Supplementary material for: Diagnostic accuracy of the interferon-gamma release assay in acquired immunodeficiency syndrome patients with suspected tuberculosis infection: a meta-analysis
Source: Infection. 2022 Mar 6;50(3):597–606. doi: 10.1007/s15010-022-01789-9 (PMC9151521; doi:10.1007/s15010-022-01789-9)
Supplement: Supplementary file 1 — Supplementary file1 (DOCX 1451 KB) [file 15010_2022_1789_MOESM1_ESM.docx]

**Diagnostic accuracy of the interferon-gamma release assay in acquired immunodeficiency syndrome patients with suspected tuberculosis infection: A meta-analysis**

Running title: IGRA in HIV

Hao Chen^1＃^, Atsushi Nakagawa^2＃^, Mikio Takamori^3＃^, Seitarou Abe^4＃^, Daisuke Ueno^5＃^, Nobuyuki Horita^6＃^, Seiya Kato^7＃^, Nobuhiko Seki^1＃^

^1^ Department of Internal medicine, Teikyo University Graduate School of Medicine, Tokyo, Japan

^2^ Department of Respiratory, Kobe City Medical Center General Hospital, Kobe, Japan

^3^ Department of Respiratory, Tokyo Metropolitan Tama Medical Center, Tokyo, Japan

^4^ Department of Respiratory, Niigata Prefectural Shibata Hospital, Niigata, Japan

^5^ Department of Emergency Medicine, Kawasaki Medical School Hospital, Okayama, Japan

^6^ Department of Pulmonology, Yokohama City University, Yokohama, Japan

^7^ Research Institute of Tuberculosis, Japan Anti-Tuberculosis Association, Tokyo, Japan

^＃^ All authors contributed equally to this article.

Corresponding author: Nobuhiko Seki

Department of Oncology, Teikyo University Hospital

2-11-1 Kaga, Itahashi, Tokyo 173-8606, Japan

E-mail: nseki@med.teikyo-u.ac.jp

Tel: 03-3964-1211; Fax: 03-3964-1211

**Supplementary Figures**

Figure S1. Flow diagram of this study

Figure S2. Forest plot of all enrolled studies including test accuracy in active TB

Pooled sensitivity and specificity are 0.66 (95%CI 0.63, 0.68) and 0.92 (95%CI 0.91, 0.93), respectively.

Figure S3. Forest plot of all enrolled studies including LTBI test accuracy

Pooled sensitivity is 0.64 (95%CI 0.61, 0.66), and specificity is not estimable.

Figure S4. Forest plot of all enrolled studies including QFT test accuracy in active TB

Pooled sensitivity and specificity are 0.66 (95%CI 0.63, 0.69) and 0.91 (95%CI 0.90, 0.92), respectively.

Figure S5. Forest plot of all enrolled studies including T-SPOT test accuracy in active TB

Pooled sensitivity and specificity are 0.65 (95%CI 0.62, 0.68) and 0.93 (95%CI 0.92, 0.94), respectively.

Figure S6. Forest plot of all enrolled studies including QFT test accuracy in LTBI

Pooled sensitivity and specificity are 0.66 (95%CI 0.56, 0.70)

Figure S7. Forest plot of all enrolled studies including T-SPOT test accuracy in LTBI

Pooled sensitivity and specificity are 0.60 (95%CI 0.56, 0.64)

Figure S8. Selection bias of studies
